# Clinical characteristics of different subtypes of adenomyosis in infertility

**DOI:** 10.1097/MD.0000000000049441

**Published:** 2026-06-26

**Authors:** MeiXian Wang, Ning Li, JingShi Wang, YiYong Liu, Lei Wang, XiaoGuang Shao

**Affiliations:** a Graduate School of Dalian Medical University, Dalian Medical University, Dalian, Liaoning, China; b Reproductive Center, Affiliated Zhongshan Hospital of Dalian University, No. 6 Jiefang Street, Zhongshan District, Dalian, Liaoning, China; c Dalian Women and Children’s Medical Group, Dalian, Liaoning, China.

**Keywords:** adenomyosis, infertility, magnetic resonance imaging

## Abstract

This study aimed to compare the clinical histories and clinicopathological characteristics between internal and external adenomyosis (ADM) among infertile women. A total of 759 infertile patients aged 24 to 40 years were enrolled from the Sports New City Branch of Dalian Maternal and Child Health Hospital (Group) from June 2018 to September 2024. All patients were divided into 3 groups according to magnetic resonance imaging-based ADM typing: internal ADM group (n = 418), external ADM group (n = 113), and non-ADM control group (n = 228). Baseline clinical histories and clinicopathological parameters were statistically compared among the 3 groups. Significant differences in clinical historical features were identified among different ADM subtypes. A history of intrauterine surgery was verified as an independent risk factor for internal ADM, while endometriosis was an independent risk factor for external ADM. Subtype-specific disparities were also observed in clinicopathological profiles. Endometritis was an independent clinicopathological feature of internal ADM, whereas posterior myometrial thickening, elevated serum carbohydrate antigen 125 levels, and decreased anti-müllerian hormone levels were independent clinicopathological characteristics of external ADM. Internal and external ADM subtypes present distinct clinical histories and clinicopathological features, indicating fundamental phenotypic differences between the 2 subtypes in infertile populations.

## 1. Introduction

Adenomyosis (ADM) is characterized by the ectopic invasion of endometrial epithelial and stromal cells into the uterine myometrium, triggering reactive fibrosis, smooth muscle hyperplasia, and myometrial hypertrophy. This condition is closely associated with female infertility, abnormal uterine bleeding, and recurrent pelvic pain.^[1,2]^ Although histopathological examination remains the diagnostic gold standard for ADM^,[3]^ advances in magnetic resonance imaging (MRI) and transvaginal ultrasound have facilitated accurate noninvasive diagnosis of ADM in clinical practice.^[4–10]^ Such imaging modalities are particularly indispensable for infertile patients, for whom invasive histological confirmation is rarely feasible. Unlike conventional histopathological evaluation, MRI enables precise delineation of layered anatomical structures within the uterine myometrium, supporting lesion location-based morphological classification. This layered anatomical partition of the uterus in reproductive-aged women was first proposed by Hricak et al^[11]^ in 1983. On T2-weighted MRI sequences, the innermost hyperintense layer corresponds to the endometrium, the outer medium-intensity region represents the outer myometrium, and the intermediate hypointense zone is defined as the inner myometrium. The inner and outer myometrial layers derive from distinct embryonic origins and exhibit heterogeneous responsiveness to female hormones.^[12]^

Kishi et al^[13]^ categorized adenomyotic lesions into 4 subtypes according to anatomical location. Subtype I lesions are confined to the inner myometrium without outer myometrial involvement; subtype II lesions are restricted to the outer myometrium while sparing the inner junctional zone; subtype III refers to isolated lesions with intact endometrial and serosal layers; and all lesions inconsistent with the above 3 categories are defined as subtype IV. Multiple subsequent studies have further validated the clinical application value of location-based ADM classification^.[14–22]^

Despite improved imaging diagnostic accuracy for ADM, the correlations between imaging-defined ADM subtypes, clinical phenotypes, and therapeutic outcomes remain insufficiently clarified.^[23]^ Existing relevant studies have primarily focused on surgical cohorts and confirmed notable subtype-specific clinicopathological differences. Specifically, patients with internal ADM tend to be older and have a higher prevalence of menstruation-related symptoms, while external ADM is more commonly complicated by infertility and pelvic endometriosis.^[14,21,24,25]^

The overall prevalence of ADM in infertile populations reaches 24.4%.^[26]^ However, no studies have systematically explored the clinical and clinicopathological characteristics of different location-based ADM subtypes among pure infertile cohorts. Therefore, this retrospective study enrolled infertile patients with MRI-confirmed ADM, stratified participants by lesion location, and comprehensively compared clinical histories and phenotypic parameters to clarify the unique clinical profiles of internal and external ADM in infertile women.

## 2. Materials and methods

### 2.1. Study subjects

A total of 759 infertile patients aged 24 to 40 years were recruited from the Sports New City Branch of Dalian Maternal and Child Health Hospital (Group) between June 2018 and September 2024. Participants were divided into an internal ADM group, an external ADM group, and a non-ADM control group based on MRI diagnostic results.

**Figure 1.**
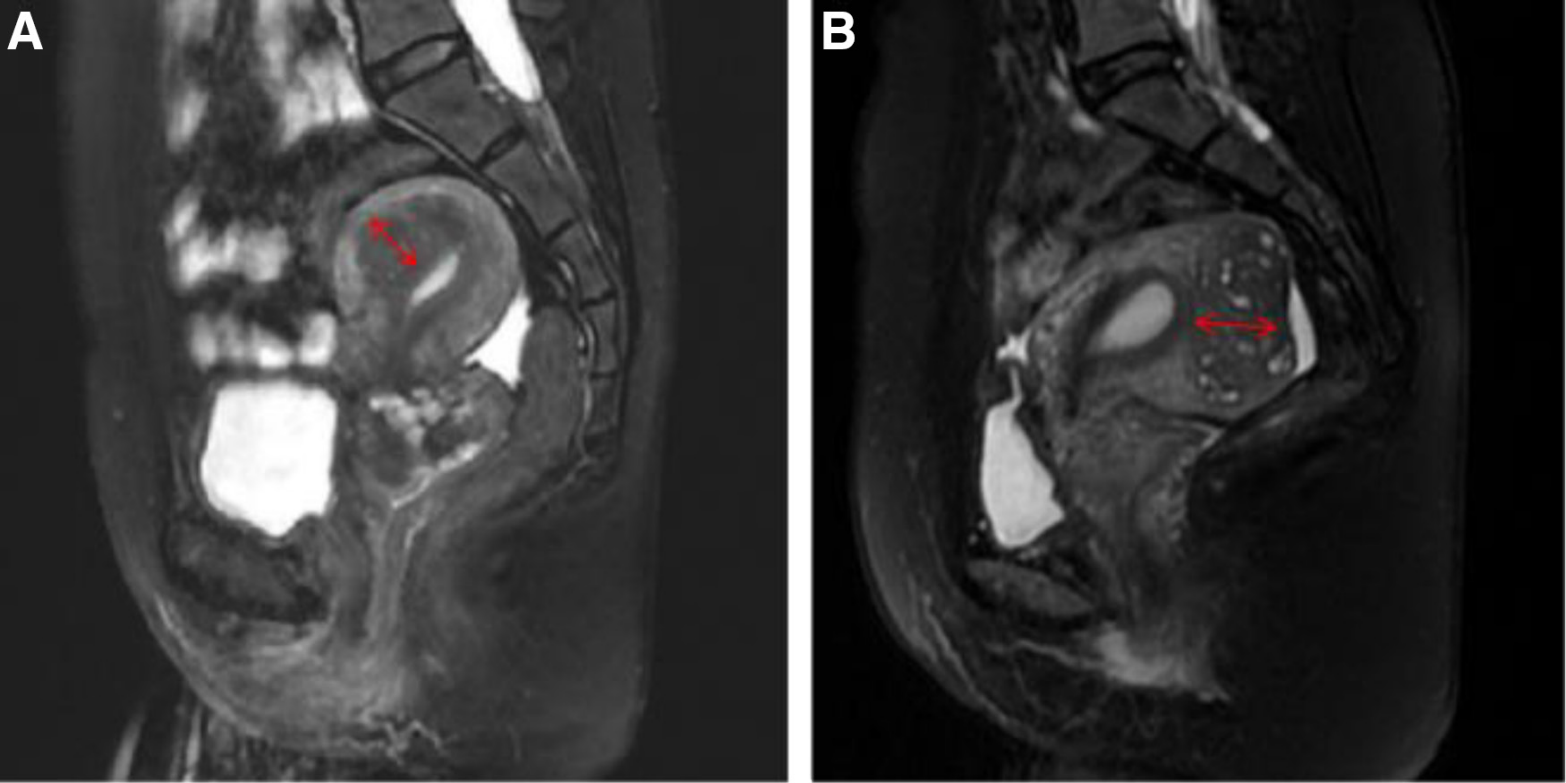
T2 - weighted MRI images of the internal and external ADM. (A) Internal ADM: The maximum junction zone thickness is ≥ 12 mm, and the ratio of the maximum junction to the ipsilateral myometrial thickness is > 40% (red arrow). The outer myometrium is normal. (B) External ADM: The lesion is located in the outer myometrium (red arrow), and there is healthy muscle tissue between the lesion and the junction zone. ADM = adenomyosis, MRI = magnetic resonance imaging.

Patients were excluded according to the following criteria: ADM with undefined lesion location; prior history of ADM-related surgery or interventional therapy; age over 40 years; congenital uterine malformation; coexisting type 0 to III uterine fibroids with a diameter > 4 cm; concurrent autoimmune diseases; chromosomal abnormalities; and incomplete key clinical and cycle data. The detailed patient screening process is illustrated in Figure 1.

### 2.2. MRI diagnostic methods and grouping

All participants underwent pelvic MRI examination using a 1.5-T superconducting MRI system (GE Healthcare) during the late follicular phase of the natural menstrual cycle.^[27]^ Sagittal, transverse, and coronal comprehensive scans were routinely performed. All MRI images were independently evaluated by 2 senior radiologists to determine ADM lesion classification based on anatomical features. In cases of diagnostic disagreement, a third experienced radiologist performed a reevaluation. Only cases with consistent diagnostic conclusions by at least 2 radiologists were included in the analysis, while inconsistent cases were excluded.

On T2-weighted sequences, maximum junction zone thickness, minimum junction zone thickness, and myometrial thickness were measured. Uterine length and anteroposterior diameter were assessed on mid-sagittal planes, and uterine transverse diameter was measured on transverse planes.

ADM was diagnosed using combined direct and indirect MRI criteria. The direct criterion was the presence of 2 to 7 mm hyperintense foci within the uterine myometrium. The indirect criterion was defined as a maximum junction zone thickness of ≥ 12 mm, plus at least one of the following auxiliary indicators: a ratio of maximum junction zone thickness to ipsilateral myometrial thickness > 40%; a junctional zone thickness difference ≥ 5.5 mm.

Location-based ADM classification was implemented as follows: internal ADM was defined as hyperintense lesions located within or adjacent to the junctional zone, surrounded by normal myometrial tissue; external ADM referred to lesions confined to the outer myometrium, with an intact junctional zone and normal muscle tissue separating lesions from the endometrium^.[16,19,24]^ Representative T2-weighted MRI images of internal and external ADM are presented in Figure 1.

This study was approved by the Institutional Ethics Committee (Batch No. 2024009).

### 2.3. Case data collection

All clinical data were retrospectively extracted from electronic medical records. Collected clinical historical variables included age, body mass index, infertility duration, pregnancy and childbirth history, ADM-related medical history, and surgical history. Clinicopathological parameters comprised menstrual status, serum carbohydrate antigen 125 (CA125) levels, uterine cavity length, uterine diameter parameters (length, anteroposterior, transverse), anterior and posterior myometrial thickness, natural-cycle endometrial thickness, causes of infertility, and ovarian reserve.

### 2.4. Evaluation criteria

- Menorrhagia: Menstrual blood volume was assessed via the pictorial blood assessment chart, with a total score ≥ 100 defined as menorrhagia.^[28]^
- Dysmenorrhea severity: A visual analog scale (VAS) was applied for pain evaluation, and a VAS score ≥ 7 was classified as severe dysmenorrhea.^[29]^
- Uterine surgery history: Included myomectomy, hysteroscopic surgery, and cesarean section.
- Intrauterine surgery history: Included hysteroscopic operation, induced abortion, uterine curettage, diagnostic curettage, and intrauterine device insertion and removal.
- Thin endometrium: Defined as endometrial thickness < 7 mm during in vitro fertilization treatment cycles.^[30,31]^
- Recurrent miscarriage: Refers to consecutive embryonic or fetal loss before 28 weeks of gestation, including recurrent biochemical pregnancy.^[32]^
- Endometriosis: Diagnosed via pelvic ultrasonography, MRI, or surgical exploration confirming ovarian or pelvic endometriotic lesions.
- Uterine volume: Calculated using the elliptical volume formula: V = 0.523 × a × b × c, where a, b, and c represent uterine length, anteroposterior diameter, and transverse diameter, respectively.^[33]^
- Chronic endometritis: For all cases of chronic endometritis, hysteroscopic endometrial biopsy identified stromal plasma cell infiltration with positive cluster of differentiation 138 staining.^[34]^
- Uterine cavity length: Measured using an artificial insemination tube during the luteal phase of the natural cycle, defined as the distance from the external cervical os to the uterine fundus.

### 2.5. Statistical methods

Statistical analysis was performed using SPSS 27.0 (IBM Corporation, Armonk). No missing data were present in the study variables, so no imputation methods were applied. The Shapiro–Wilk test was utilized to evaluate the normality of continuous variables. Continuous data with a non-normal distribution were expressed as medians with interquartile ranges [M (P_25_, P_75_)], whereas categorical variables were presented as frequencies and percentages (%). For continuous variables that did not conform to a normal distribution, the Kruskal–Wallis test was employed for multi-group comparisons, followed by post hoc pairwise Mann–Whitney *U* tests with the Bonferroni correction for multiple testing. Categorical data were compared using the chi-square test or Fisher exact test as appropriate.

Binary logistic regression analysis was performed to screen independent factors associated with internal and external ADM. Variables exhibiting significant differences in univariate analyses of baseline clinical and historical characteristics were enrolled in the multivariate regression models. Odds ratios and 95% confidence intervals were calculated for each predictive factor. A 2-tailed *P* value < .05 was defined as statistically significant.

## 3. Results

### 3.1. Baseline characteristics of different ADM subtypes

**Figure 2.**
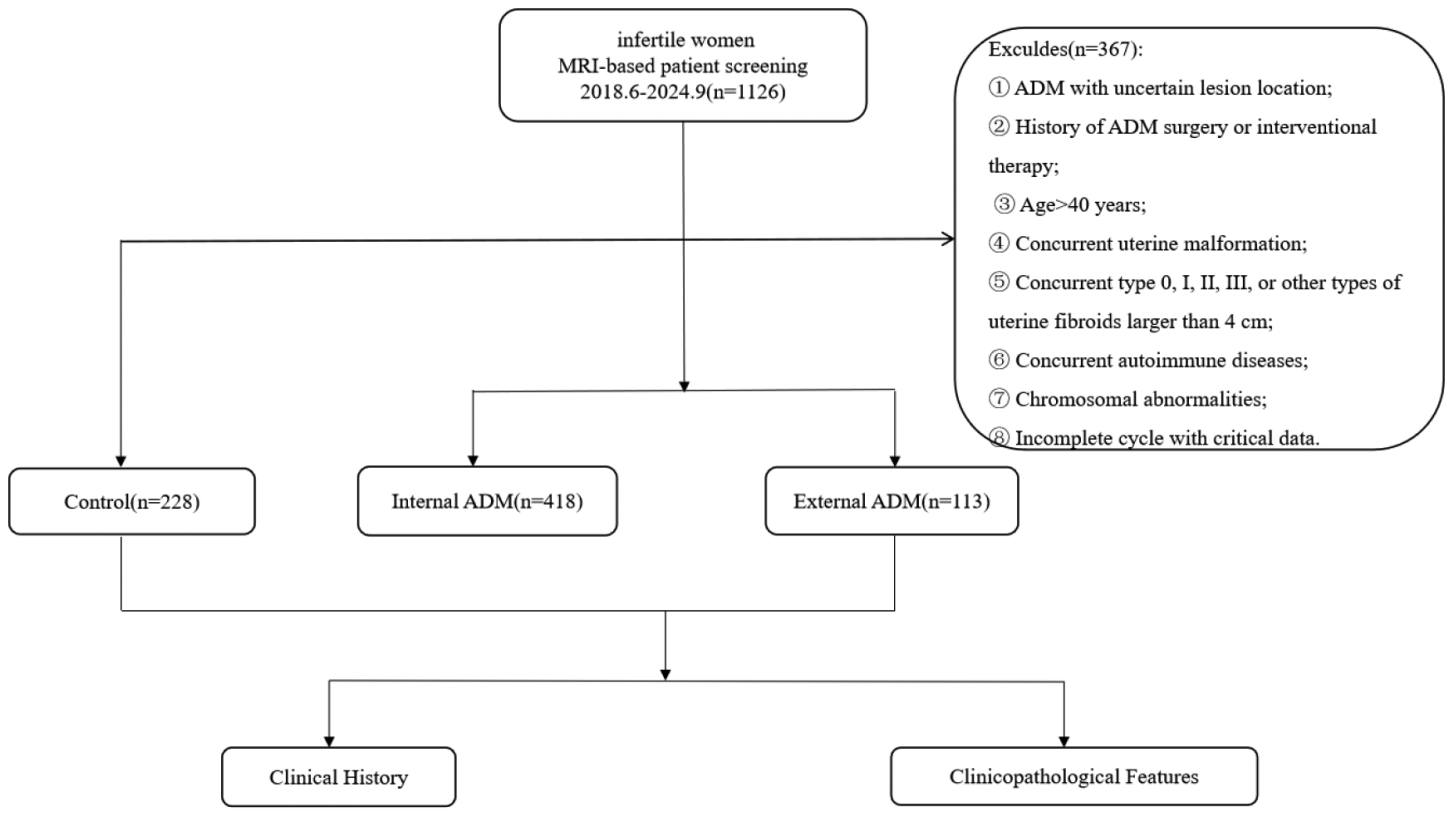
Flowchart of the study group. ADM = adenomyosis, MRI = magnetic resonance imaging.

A total of 1126 infertile women undergoing pelvic MRI were preliminarily screened between June 2018 and September 2024. After excluding 367 ineligible cases, 759 patients were finally enrolled, including 481 internal ADM cases, 113 external ADM cases, and 228 non-ADM controls (Fig. 2).

Baseline analysis showed no significant intergroup differences in age, body mass index, and infertility duration among the 3 groups (Table 1).

**Table 1:** Baseline characteristics of different ADM subtypes.

Observation index	Control (n = 228)	Internal ADM (n = 418)	External ADM (n = 113)	*P* value
Age (yrs)	34 (32–37)	34 (31–36)	34 (32–37)	.165
BMI (kg/m^2^)	22.75 (21.30–24.70)	22.70 (21.10–25.40)	22.18 (20.70–24.41)	.142
Duration of infertility (years)	3 (2–5)	3 (2–5)	3 (1.5–4)	.178

Data are expressed as median (25th percentile, 75th percentile).

ADM = adenomyosis, BMI = body mass index.

*P* values were determined using the Kruskal–Wallis *H* test.

### 3.2. Comparison of clinical histories of different ADM subtypes

Significant intergroup differences were observed in the proportion of primary infertility, the number of abortions, history of intrauterine adhesions, history of endometriosis, history of intrauterine surgery, and the number of intrauterine surgeries (Table 2). Pairwise post hoc analysis revealed that the external ADM group had a significantly higher rate of primary infertility and a history of endometriosis. The internal ADM group exhibited a higher incidence of intrauterine adhesions and more histories of intrauterine surgery compared with the control group. No significant differences were found in parity history, ectopic pregnancy, recurrent miscarriage, or history of uterine surgery across groups.

**Table 2:** Clinical history parameters of different ADM subtypes.

Observation index	Control (n = 228)	Internal ADM (n = 418)	External ADM (n = 113)	*P* value
Reproductive history				
Primary infertility, n (%)	107 (46.93)	204 (48.80)	74 (65.49)*,†	.008
One prior abortion, n (%)	85 (37.28)	114 (27.27)*	29 (25.66)*	< .001
≥ 2 prior abortions, n (%)	26 (11.40)	100 (23.92)*	10 (8.85)†	< .001
Parity history, n (%)	22 (9.65)	35 (8.37)	5 (4.42)	.246
Ectopic pregnancy, n (%)	13 (5.70)	26 (6.22)	7 (6.19)	.767
Recurrent miscarriage, n (%)	5 (2.19)	25 (5.98)	6 (5.31)	.092
Uterine disease and surgical history				
Intrauterine adhesion, n (%)	3 (1.32)	32 (7.66)*	5 (4.42)	.002
Endometriosis, n (%)	4 (1.75)	40 (9.57)*	36 (31.86)*,†	< .001
Uterine surgery, n (%)	14 (6.14)	41 (9.81)	12 (10.62)	.188
Intrauterine surgery, n (%)	72 (31.58)	249 (59.57)*	36 (31.86)†	< .001
Number of intrauterine surgeries	0 (0,1)	1 (0,1)*	0 (0,1)†	< .001

Categorical data are expressed as n (%); continuous data are expressed as median (25th percentile, 75th percentile). Overall intergroup comparisons were conducted using the *χ*^2^ test for categorical variables and the Kruskal–Wallis H test for continuous variables. Bonferroni-corrected post hoc pairwise comparisons were performed if the overall *P* < .05.

ADM = adenomyosis, n = number of patients.

* *P* < .05, vs control group.

† *P* < .05, vs internal ADM group.

### 3.3. Multivariate logistic regression analysis of clinical historical characteristics

Variables with significant univariate differences (primary infertility, ≥ 2 prior abortions, endometriosis history, and intrauterine surgery history) were included in the multivariate logistic regression model.

Covariate multicollinearity assessment showed tolerance values ranging from 0.619 to 0.968 and variance inflation factor values from 1.033 to 1.614, indicating no obvious multicollinearity.

The Hosmer–Lemeshow test verified good model fitness (*P* > .05).

**Figure 3.**
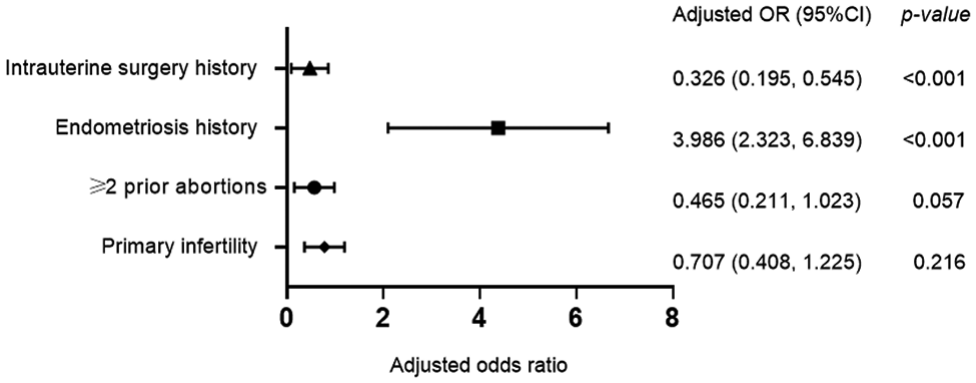
Multivariate logistic regression analysis of clinical history in different ADM subtypes. ADM = adenomyosis, CI = confidence interval, OR = odds ratio.

The regression results demonstrated that a history of intrauterine surgery was an independent correlative factor for internal ADM, whereas endometriosis history was independently associated with external ADM (Fig. 3).

### 3.4. Comparison of clinicopathological features of different ADM subtypes

No significant intergroup differences were detected in menstrual characteristics, endometrial thickness, and infertility etiology distribution. In contrast, significant disparities were observed in VAS pain score, severe dysmenorrhea rate, thin endometrium prevalence, endometritis, uterine fibroids, serum CA125 level, uterine cavity length, uterine volume, and anteroposterior myometrial thickness (Table 3).

**Table 3:** Clinicopathological and ovarian reserve characteristics of different ADM subtypes.

Observation index	Control (n = 228)	Internal ADM (n = 418)	External ADM (n = 113)	*P* value
Menstrual cycle				
Cycle length < 21 d, n (%)	5 (2.19)	4 (0.96)	3 (2.65)	.296
Cycle length 21–35 d, n (%)	200 (87.72)	351 (83.97)	98 (86.73)	.400
Cycle length > 35 d, n (%)	23 (10.09)	63 (15.07)	12 (10.62)	.114
Cycle duration				.533
≤ 7 d, n (%)	220 (96.49)	405 (96.89)	107 (94.69)	-
>7 d, n (%)	8 (3.51)	13 (3.11)	6 (5.31)	-
Menorrhagia, n (%)	0 (0.00)	2 (0.48)	1 (0.88)	.435
VAS score	0 (0,2)	0 (0,0)	2 (0,4)*,†	< .001
Severe dysmenorrhea, n (%)	0 (0.00)	8 (1.91)	14 (12.39)*,†	< .001
Thin endometrium, n (%)	3 (1.32)	25 (5.98)*	1 (0.88)†	.003
Endometritis, n (%)	13 (5.70)	99 (23.68)*	9 (7.96)†	< .001
Uterine fibroids, n (%)	30 (13.16)	75 (17.94)	38 (33.63)*,†	< .001
CA125 (U/L)	17.17 (12.11–21.64)	19.80 (12.80–29.61)*	42.53 (24.03–68.67)*,†	< .001
Uterine cavity length (cm)	7.5 (7.0–8.0)	8.0 (7.0–8.0)*	7.8 (7.5–8.0)	< .001
Uterine body volume (mL)	54.83 (51.74–58.29)	78.24 (70.29–91.64)*	81.94 (70.11–97.19)*	< .001
Anterior uterine myometrium thickness (mm)	16.38 (15.32–18.36)	19.00 (16.19–24.75)*	16.36 (15.36–18.36)†	< .001
Posterior uterine myometrium thickness (mm)	18.68 (16.47–21.36)	26.36 (22.08–29.31)*	30.01 (28.21–35.36)*,†	< .001
Endometrial thickness (mm)	9.0 (8.2–10.0)	9.0 (8.0–11.0)	9.1 (8.5–10.1)	.417
Causes of Infertility				
Pelvic and fallopian tube factors, n (%)	130 (57.0)	243 (58.1)	72 (63.7)	.474
Ovulatory dysfunction, n (%)	40 (17.5)	76 (18.2)	14 (12.4)	.343
Male factors, n (%)	43 (18.9)	74 (17.7)	19 (16.8)	.885
Other causes, n (%)	15 (6.6)	25 (6.0)	8 (7.1)	.897
Ovarian Reserve				
AMH (ng/mL)	2.5 (1.4–4.2)	2.5 (1.3–4.4)	1.7 (0.8–2.7)*,†	< .001
AFC	10 (7–18)	12 (7–19)	8 (5–13)*,†	< .001

Categorical variables are expressed as n (%); continuous variables are presented as median (25th percentile, 75th percentile). Intergroup overall comparisons were performed using *χ*^2^ test for categorical data and Kruskal–Wallis H test for continuous data. Bonferroni-adjusted post hoc pairwise comparison was conducted when overall *P* < .05.

ADM = adenomyosis, AMH = anti-Müllerian hormone, AFC = antral follicle count, CA125 = carbohydrate antigen 125, d = days, n = number of patients, VAS = visual analog scale.

* *P* < .05, vs Control group.

† *P* < .05, vs Internal ADM group.

The internal ADM group had a higher prevalence of thin endometrium and endometritis, as well as thicker anterior myometrium. The external ADM group presented with higher VAS scores, a higher proportion of severe dysmenorrhea and uterine fibroids, elevated serum CA125 levels, and increased posterior myometrial thickness. Both ADM subtypes had significantly enlarged uterine volume compared with the control group, with no statistical difference between the 2 ADM groups. In addition, patients with external ADM exhibited significantly impaired ovarian reserve function.

### 3.5. Multivariate logistic regression analysis of clinicopathological characteristics

To ensure model stability, subjective indicators and collinear variables (dysmenorrhea parameters, anterior myometrial thickness, and uterine fibroids) were excluded.

Finally, serum CA125 level, anti-Müllerian hormone (AMH), antral follicle count, thin endometrium rate, endometritis rate, and posterior myometrial thickness were incorporated into the regression model.

Covariate multicollinearity assessment showed tolerance values ranging from 0.309 to 0.957 and variance inflation factor values from 1.045 to 3.237, indicating no obvious multicollinearity. The Hosmer–Lemeshow test verified good model fitness (*P* > .05).

**Figure 4.**
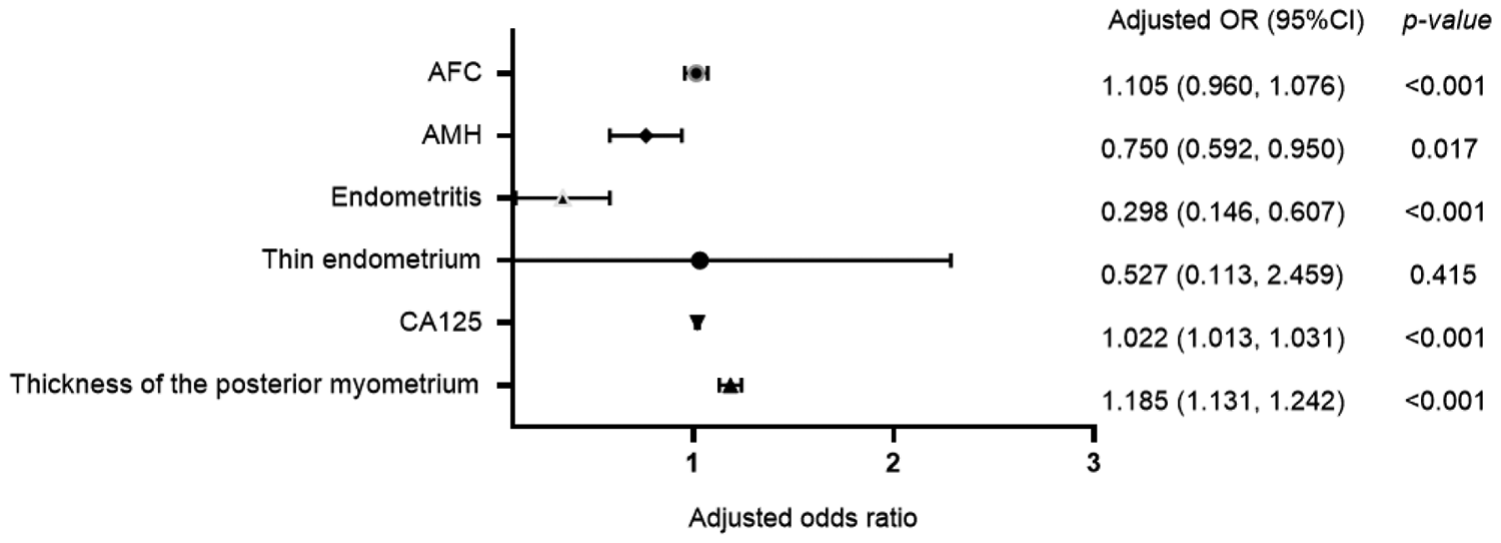
Multivariate logistic regression analysis of clinical characteristics in different ADM subtypes ADM = adenomyosis, AFC = antral follicle count, AMH = anti-müllerian hormone, CA125 = carbohydrate antigen 125.

Multivariate analysis identified endometritis as an independent clinicopathological correlate of internal ADM. For external ADM, posterior myometrial thickening, elevated serum CA125 levels, and reduced AMH levels were independent phenotypic characteristics (Fig. 4).

## 4. Discussion

The present study confirmed significant differences in clinical historical profiles and clinicopathological phenotypes between internal and external ADM among infertile populations. A history of intrauterine surgery was independently correlated with internal ADM, while endometriosis comorbidity was an independent associative factor for external ADM. In terms of clinicopathological features, endometritis was a unique independent characteristic of internal ADM, whereas posterior myometrial thickening, elevated CA125 levels, and decreased AMH levels were typical independent manifestations of external ADM.

Our findings are consistent with previous evidence derived from surgical cohorts. Bourdon et al^[24]^ analyzed 109 internal ADM and 78 external ADM surgical cases and verified that uterine surgery history was closely related to internal ADM, while endometriosis was associated with external ADM. Liang et al^[25]^ further enrolled 77 internal and 54 external ADM surgical patients and found that low educational level, high parity, and repeated uterine curettage were correlated with internal ADM, while ovarian and deep infiltrating endometriosis were predominant in external ADM. Despite differences in enrolled populations (surgical cohorts in previous studies vs infertile cohort in the present study), the consistent subtype-specific risk factor differentiation across studies supports divergent pathogenetic backgrounds between the 2 ADM subtypes.

Accumulated morphological and molecular evidence further supports such phenotypic and pathogenetic discrepancies. Kishi et al^[35]^ reported distinct fibrosis-related protein expression patterns according to ADM lesion location, proposing that internal ADM mainly originates from the direct invasion of eutopic endometrium into the inner myometrium, while external ADM is more likely secondary to the infiltration of ectopic endometrial cells associated with pelvic endometriosis. Khan et al^[36]^ found that external ADM shared highly consistent histological morphology with deep infiltrating endometriosis, whereas internal ADM resembled normal endometrial tissue, further verifying subtype-specific pathogenetic features. Chapron et al^[19]^ also confirmed a definite association between outer myometrial lesions and deep infiltrating endometriosis. Although multiple hypotheses, including endometrial invagination, metaplasia, and endometrial–myometrial disruption^,[2,37–40]^ have been proposed to interpret ADM pathogenesis, the exact molecular mechanisms remain incompletely clarified and require further exploration.

We further analyzed typical menstrual and pain symptoms across ADM subtypes. No significant difference in menorrhagia incidence was observed between internal and external ADM groups in our infertile cohort, which differs from previous surgical-based studies that reported a higher menorrhagia rate in internal ADM.^[24,25]^ For dysmenorrhea, internal ADM patients presented with mild pain symptoms comparable to non-ADM infertile individuals, while external ADM was characterized by severe dysmenorrhea. This finding was consistent with Liang et al,^[25]^ whereas Bourdon et al^[24]^ detected no inter-subtype difference in dysmenorrhea severity. Such inter-study inconsistencies can be reasonably attributed to discrepancies in enrolled population types and ADM lesion severity.

The external ADM group had a higher proportion of primary infertility, which is consistent with previous reports^.[24,25]^ No significant difference in infertility etiology composition was found between the 2 subtypes, indicating that ADM anatomical classification does not alter the overall infertility etiology distribution in affected patients. In terms of ovarian reserve, external ADM patients presented with poorer ovarian function than internal ADM and non-ADM individuals. The high comorbidity of endometriosis in external ADM may contribute to impaired ovarian reserve, which provides a reasonable explanation for the reduced AMH levels observed in the external ADM subgroup.

From a phenotypic perspective, internal ADM lesions are contiguous with the native endometrium, which increases the susceptibility of the uterine cavity to intrauterine pathological changes, including endometrial thinning and endometritis. In contrast, external ADM is closely correlated with invasive pelvic endometriosis,^[4,26,27]^ which is associated with its typical clinical manifestations, such as posterior myometrial thickening and elevated serum CA125 levels. Notably, internal ADM in infertile populations presents with atypical and inconspicuous clinical symptoms and laboratory abnormalities; thus, its diagnosis primarily relies on imaging examinations rather than clinical manifestations.

Several notable limitations of the present study should be acknowledged. First, endometriosis in our cohort was diagnosed based on a combination of surgical exploration and pelvic ultrasonography. Unified intraoperative pathological grading data were unavailable for all participants, making precise quantitative assessment of endometriosis severity and anatomical subtypes unfeasible in this retrospective analysis. Second, the relatively small sample size of the external ADM subgroup may compromise the statistical power and stability of the multivariate regression model. Third, this single-center retrospective observational study may introduce residual confounding factors and potential statistical bias. Most importantly, consistent with the inherent limitations of retrospective research, our findings only demonstrate clinical correlative differences between ADM subtypes, rather than confirming definite causal relationships or inherent biological specificities, which may restrict result interpretation. Future multicenter, large-sample prospective studies are warranted to validate our conclusions and minimize statistical bias. In addition, longitudinal cohort studies integrating pregnancy outcomes will help further explore the prognostic value of ADM anatomical typing and improve its translational applicability in reproductive clinical practice.

## 5. Conclusions

In summary, distinct ADM lesion locations are closely correlated with differential clinical histories and clinicopathological phenotypes in infertile populations, indicating obvious phenotypic heterogeneity and divergent pathogenetic characteristics between internal and external ADM subtypes.

## Acknowledgments

The authors thank the staff of the Department of Dalian Women and Children’s Central Hospital for their cooperation and support of this study.

## Author contributions

**Data curation:** Ning Li, JingShi Wang, YiYong Liu.

**Formal analysis:** Lei Wang.

**Writing – original draft:** MeiXian Wang.

**Writing – review & editing:** XiaoGuang Shao.
